# The Enigma of Low COVID-19 Fatality Rate in India

**DOI:** 10.3389/fgene.2020.00854

**Published:** 2020-07-28

**Authors:** Arghadip Samaddar, Ravisekhar Gadepalli, Vijaya Lakshmi Nag, Sanjeev Misra

**Affiliations:** ^1^Department of Microbiology, All India Institute of Medical Sciences, Jodhpur, India; ^2^Department of Surgical Oncology, All India Institute of Medical Sciences, Jodhpur, India

**Keywords:** COVID-19, SARS-CoV-2, Indian population, angiotensin converting enzyme 2, killer cell immunoglobulin-like receptors, microRNA

## Abstract

Coronavirus disease 2019 (COVID-19), an acute onset pneumonia caused by a novel *Betacoronavirus* Severe Acute Respiratory Syndrome Coronavirus 2 (SARS-CoV-2) has rapidly evolved into a pandemic. Though its origin has been linked to the Wuhan City of China’s Hubei Province in December 2019, recent reports claim that the original animal-to-human transmission of the virus probably happened sometime between September and October 2019 in Guangdong Province, rather than Hubei. As of July 3, 2020, India has reported a case positivity rate of 6.5% and a fatality rate of 2.8%, which are among the lowest in the world. Also, the severity of the disease is much less among Indians as evidenced by the low rate of ICU admission (15.3%) and the need for mechanical ventilation (4.16%). As per the World Health Organization (WHO) situation report 165 on July 3, 2020, India has one of the lowest deaths per 100,000 population (1.32 deaths against a global average of 6.04). Several factors related to the pathogen, host and environment might have some role in reducing the susceptibility of Indians to COVID-19. These include some ongoing mutations that can alter the virulence of the circulating SARS-CoV-2 strains, host factors like innate immunity, genetic diversity in immune responses, epigenetic factors, genetic polymorphisms of ACE2 receptors, micro RNAs and universal BCG vaccination, and environmental factors like high temperature and humidity which may alter the viability and transmissibility of the strain. This perspective -highlights the potential factors that might be responsible for the observed low COVID-19 fatality rate in Indian population. It puts forward several hypotheses which can be a ground for future studies determining individual and population susceptibility to COVID-19 and thus, may offer a new dimension to our current understanding of the disease.

## Introduction

In December 2019, an outbreak of fatal pneumonia, the Coronavirus Disease 2019 (COVID-19), caused by a novel *Betacoronavirus*, Severe Acute Respiratory Syndrome Coronavirus 2 (SARS-CoV-2) was reported from the Wuhan City of China’s Hubei Province. The outbreak was declared as a Public Health Emergency of International Concern by the World Health Organization (WHO) on January 30, 2020 and a global pandemic on March 11, 2020.^1^ ^,2^ Though its origin has been linked to Hubei Province, recent phylogenetic analysis claims that the original animal-to-human transmission of the virus probably happened some 3 to 4 months before December 2019 in Guangdong Province, rather than Hubei (Forster et al., 2020). As of July 3, 2020, the disease has affected more than 10.7 million people across 216 countries and territories with 517,877 deaths.^3^ A difference in the case fatality rates (CFR) was observed across countries, possibly due to demographic variations, differences in the virus strains in circulation and the nature of containment measures implemented. The fatality rates in the United States, United Kingdom, Italy, France, and Spain have surpassed that of China by manifolds. India, the second most populous country in the world, with a population of 1.3 billion people, had documented the first case of COVID-19 on January 30, 2020, the same day as Italy (Kaushik et al., 2020). As of July 3, 2020, India has reported a total of 625,544 cases and 18,213 deaths which reflects a milder trajectory with lower case positivity (6.5%) and fatality (2.8%) rates compared to the global figures.^3^

The traditional model for any infectious disease consists of a triad of the causative agent, the host, and an environment in which the agent and host are brought together, causing the disease to occur in the host. Little is known regarding the origin of SARS-CoV-2. There have been speculations that the virus got transmitted to humans from bats or pangolins, but conclusive evidence regarding the same is lacking. Genomic sequence data indicate that SARS-CoV-2 has 96.2% sequence homology with a bat coronavirus RaTG13 (Zheng, 2020), and 91% homology with a pangolin coronavirus (Zhang et al., 2020). Besides, it also shares 79.5% identity with SARS-CoV (Zheng, 2020). A recent study suggests that pangolins are the natural reservoirs of SARS-CoV-2-like coronavirus, with the Pangolin-CoV having a more closely related S1 protein to that of SARS-CoV-2 (Zhang et al., 2020). It is unclear how the animal–to-human transmission occurred and whether the virus acquired greater pathogenic potential and transmissibility after having entered the human host. More studies need to be focussed in this aspect for better understanding of the pathogenicity. The pandemic has made a considerable impact on health-care infrastructure worldwide, with medical facilities struggling to cope-up with the increased demands for life-saving medicines, ventilators and personal protective equipments. Despite the catastrophic consequences of this pandemic on several affluent nations, its impact on Indian population seems to be much lower both in terms of severity as well as CFR. Of the total 227,439 active cases of COVID-19 till July 03, 2020 in India, 15.3% required ICU admission, 4.16% required ventilator support and 15.9% required supplemental oxygen, all of which point toward a less severe disease among Indians.^4^ So what is that “X” factor apparently safeguarding the Indian population from the wrath of this pandemic? This perspective provides an insight into the potential factors that might be responsible for the observed low COVID-19 severity and fatality rate in Indian population and illuminates the areas of future research which may help in understanding the disease process more vividly.

### How Do the Indian SARS-CoV-2 Strains Differ From the Strains Elsewhere?

Earlier, six genera of coronaviruses were known to cause human disease, of which four (α-CoV-229E and NL63, and β-CoV- HKU1 and OC43) are responsible for mild respiratory infections, mostly in pediatric age group while two (SARS-CoV and Middle East Respiratory Syndrome coronavirus [MERS CoV]) are associated with severe outbreaks (Yang and Wang, 2020). SARS-CoV-2 is a positive sense single-stranded RNA virus harboring two major genes, open reading frame 1a (ORF1a) and ORF1b, which together encode 16 non-structural proteins (NSP1–NSP16). These NSPs are organized to form a replication–transcription complex (RTC) that is involved in transcription and replication. NSP3 and NSP5 encode for Papain-like protease (PLP) and Chymotrypsin-like protease (3CL), respectively, which help in peptide cleaving and host innate immune antagonism. NSP12 and NSP15 encode for RNA-dependent RNA polymerase (RdRp) and RNA helicase, respectively. The structural genes encode four structural proteins: spike (S), envelope (E), membrane (M), and nucleocapsid (N), and several accessory proteins (Astuti and Ysrafil, 2020).

**FIGURE 1:**
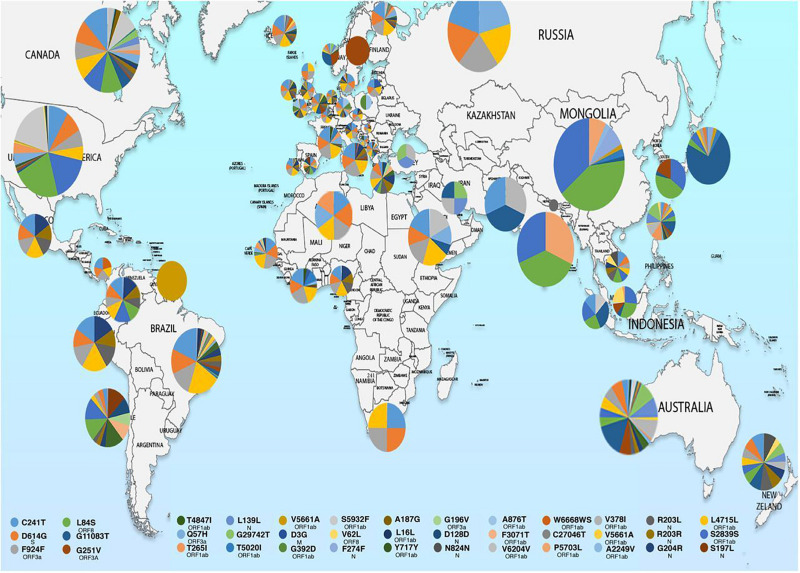
Map showing geographical distribution of SARS-CoV-2 hotspot mutations from December 24, 2019 to March 25, 2020. The pie charts show the relative frequencies of mutations in different populations. The mutational events are color coded as shown in the key. Reproduced from Laamarti et al. (2020).

Molecular characterization of SARS-CoV-2 strains based on whole-genome sequencing revealed clustering of the prototype Wuhan strain belonging to the O clade (MN908947.3/SARS-COV-2/HUMAN/CHN/Wuhan-Hu-1/2019), indicating that the virus might have originated in China and eventually spread worldwide (Chen et al., 2020; Fan et al., 2020). The Indian SARS-CoV-2 strains, unlike the strains elsewhere, are more closely related to bat-CoV RaTG13 (93% homology) than pangolin CoV (83.5% homology). However, the receptor binding domain (RBD) of S protein of Indian SARS-CoV-2 strains are more closely related to pangolin-CoV (Zhang et al., 2020). A recent study from National Institute of Cholera and Enteric Diseases, West Bengal, India analyzed the genome type clusters of 46 Indian SARS-CoV-2 isolates and observed a monophyletic clade of the virus co-existing with the prototype Wuhan strain (clade O) and clustering along with the Indian isolates, suggesting introduction of the virus in India from several countries. Phylogenetic analysis of SARS-CoV-2 genomes demonstrated two major lineages, L (leucine) and S (serine), based on single nucleotide polymorphisms (SNPs) at positions 8,782 (orf1ab: T8517C) and 28,144 (ORF8: C251T, S84L). The L lineage carries a significantly higher number of derived mutations than S lineage, indicating that these two lineages might have different rates of transmission and replication. Also, the L lineage has been described to be more aggressive and contagious than S lineage. In India, the L lineage is more prevalent (95.7%) than S lineage (4.3%) (Banerjee et al., 2020), with reports claiming higher fatality rates at places where the L type is dominant as in the Indian state of Gujarat (CFR = 5.4%, the highest in India).^5^^,^^6^ These observations indicate that strain difference has some impact on virulence and pathogenesis. Scientists from the National Institute of Biomedical Genomics, India, performed phylodynamic analyses and examined the temporal and spatial evolution of the virus in 3636 SAR-CoV-2 sequences deposited in Global Initiative on Sharing All Influenza Data (GISAID) from 55 countries and observed that at least 10 distinct clades (A1a, A2, A2a, A3, A6, A7, B, B1, B2, and B4) of the virus have originated from the ancestral clade O (Table 1). Though initially, the ancestral type was most frequent in all countries due to return of travelers from China, it has gradually been replaced by other types with clade A2a (having non-synonymous D614G mutation in the S1/S2 furin cleavage site of S protein) being the most dominant type in India and also, globally (Biswas and Majumder, 2020). Phylogenetic analysis has revealed significant regional variation in frequencies of different clades across countries. For example, in the United States, clade B1 is predominant in Washington D.C. and West Coast, while in New York and East Coast, clade A2a is the modal type (Biswas and Majumder, 2020; Brufsky, 2020). An analysis of 35 viral sequences submitted to GISAID from India revealed the existence of four clades: ancestral clade O (14.3%) and derived clades A2a (45.7%), A3 (37.1%), and B (2.9%). All individuals infected with A3 had travel history to Iran, while those with A2a had no history of international travel. Thus, a temporal decline in the diversity of SARS-CoV-2 clades has been observed globally, which may affect transmission and pathogenicity. A mutational analysis showed co-circulation of two groups (major and minor) of the mutated virus in India. The “major group” (52.2%) represents A2a clade that harbors four co-existing SNPs: 241C>T (5′ UTR), 3037C>T (F106F, NSP3), 14403C>T (P323L, RdRP/NSP12) and 23403A>G (D614G, S glycoprotein). The “minor group” (30.4%) consists of strains showing five distinct mutations: 13730C>T (A97V, RdRP/NSP12), 23929C>T (Y789Y, S), 28311C>T (P13L, N), 6312C>A (T1198K, NSP3) and 11083G>T (L37F, NSP6). All these mutations, except 11083G>T (L37F, NSP6) are unique to Indian SARS-CoV-2 strains (Guan et al., 2020; Mercatelli and Giorgi, 2020; Wang et al., 2020). Mutations 22374A>G (Q271R), 245 24933G>T (G1124V) and 22444C>T (D294D) in S gene and missense mutations Q271R and G1124V in S protein have been observed exclusively in Indian strains, which might alter the protein function and thus, affect virulence and pathogenicity (Banerjee et al., 2020). Researchers from Translational Bioinformatics Group at International Center for Genetic Engineering and Biotechnology (ICGEB) in collaboration with the Department of Biochemistry, Jamia Hamdard, New Delhi, India, performed an integrated mutational analysis of SARS-CoV-2 genomes from different geographical locations, including India, Italy, United States, Nepal and Wuhan, and observed a novel mutation in S protein (A930V, 24351C>T) of the Indian strain, which was absent in other strains (Sardar et al., 2020). A triple site mutation 28881-28883 GGG>AAC has been observed at 203/204 region of the N gene that might alter the phosphorylation of serine residue of N protein and interfere with its function, thus reducing the pathogenicity of the strain (Ayub, 2020). Besides, some Indian strains have displayed unique mutations in the NSP3 gene at positions 6310C>A (S1197R), 7392C>T (P1558L) and 6466A>G (K1249K) (Banerjee et al., 2020). The RNA-dependent RNA polymerase (RdRP)/NSP12 protein is an integral component of the viral replication machinery and any mutation in this protein might interfere with viral replication and cause accumulation of novel mutations. In Indian SARS-CoV-2 strains, two mutations: 14408C>T (P323L) and 13730C>T (A97V) in RdRP gene have been documented which might have altered the secondary structure of RdRP and resulted in simultaneous emergence of major and minor groups of the virus with characteristic co-evolving mutations (Banerjee et al., 2020). A study from University of Bologna, Italy, analyzed 10,014 SARS-CoV-2 genomic sequences in comparison with the reference Wuhan genome and revealed a low mutation rate of the virus, with an average of 6.7 mutations per sample with respect to the reference strain. According to the study, SNPs are the most common mutational events observed worldwide as well as in Asia. The most common mutation observed in Asia is G11083T, causing a SNP at position 37 of theNSP6. Several other mutations like ORF8:L84S (China) and ORF3a:G251V (Hong Kong) have also been documented, with a regional variation in the type and nature of such events (Mercatelli and Giorgi, 2020). A comparative genomic analysis of 3067 SARS-CoV-2 genomes from 59 countries revealed the presence of singleton mutations in 26 countries with United States accounting for the highest number of mutations (44% of total mutations). The mutations G251V (in ORF3a), L84S (in ORF8) and S5932F (in ORF1ab) exist in all continents except Africa. Also, the genome variability is most prominent in America, Australia and New Zealand (Laamarti et al., 2020). Figure 1 depicts the geographic and genomic distribution of SARS-CoV-2 mutations. Such genomic diversity may give rise to new clades with variations in transcription and replication rates, which in turn may affect virulence and transmissibility. Studies combining genomic details with demographic and epidemiological data are essential to identify any ongoing mutation and its impact on virulence and severity of the disease across populations.

**TABLE 1:** Phylogenetics clades of SARS-CoV-2 with their order of evolution and the defining mutation(s) (Biswas and Majumder, 2020).

Phylogenetics clade	Order of evolution	Defining mutation(s)
O	1	Ancestral clade
B	2	ORF8 – L84S
B1	3	ORF8 – L84S, nucleotide – C18060T
B2	4	ORF8 – L84S, nucleotide – C29095T
B4	5	ORF8 – L84S, N – S202N
A3	6	ORF1a – V378I, ORF1a – L3606F
A6	7	nucleotide – T514C
A7	8	ORF1a – A3220V
A1a	9	ORF3a – G251V, ORF1a – L3606F
A2	10	S – D614G
A2a	11	S – D614G, ORF1b – P314L

### Do Variations in Host Factors Determine the Susceptibility to SARS-CoV-2?

The host-pathogen interaction in COVID-19 has shown striking variations in fatality rates across different geographical regions, depending on age, smoking habits and pre-existing comorbidities (Liu et al., 2020). China, the epicenter of this pandemic, has reported an overall CFR of 5.5% among infected patients, while it is significantly lower in Singapore (0.06%), South Korea (2.2%), and Japan (5.2%). The highest CFR has been observed in France (18.9%) followed by Belgium (15.8%), Italy (14.4%), United Kingdom (14%), Spain (11.4%), Canada (8.2%), Sweden (7.7%), the United States (4.9%), Brazil (4.25%), and Germany (4.6%).^3^ India has reported only 1.32 deaths per 100,000 population against a global average of 6.04, which is among the lowest in the world.^7^ The potential host factors that might alter the susceptibility of the Indian population to SARS-CoV-2 are discussed below.

First, the Indians are exposed to an enormous microbial load early in life which confers a broad-based immunity to the population. The wide variety of pathogens, including bacteria, viruses, fungi, and parasites to which the Indians are exposed since birth, leads to induction of immune responses and also adaptive changes in cell frequencies and functions. A comparative analysis of the immune phenotypes in Indian and American newborns revealed that Indian infants had a higher proportion of dendritic cells, monocytes, natural killer (NK) cells, memory CD4 + T cells, and naïve B cells, compared to American infants (Rathore et al., 2018). Such variations in immune systems can be attributed to heritable and non-heritable influences. Heritable factors have germline inheritance and have a minor influence on inter-individual and population variation in immune responses (Brodin et al., 2015; Brodin and Davis, 2017). A population-based cohort study (ImmVar project) involving subjects of African-American, East Asian and European ancestry analyzed the variability in functional responses of T-cells and dendritic cells by gene profiling and concluded that heritable factors accounted for only 22% of the overall variation in gene expression (De Jager et al., 2015). Non-heritable factors include environmental influences, such as infections and vaccines, stochastic epigenetic changes arising from imperfect replication machinery, and symbiotic and pathogenic microbes. Non-heritable influences are the major factors determining immune variation (Brodin et al., 2015; Brodin and Davis, 2017). Brodin et al. (2015) analyzed various immunological parameters, such as cell population frequencies, cytokine responses, and serum proteins in 210 healthy twins and found that majority of these determinants are dominated by non-heritable influences, indicating that the human immune system is very much shaped by the environment and the variety of microbes that an individual encounters in their lifetime, Besides these factors, India has an overwhelming burden of tuberculosis, malaria and HIV, much higher than the developed countries. Due to such endemicity, Indians are constantly subjected to pathogen-assault, giving rise to a more proactive cell-mediated immune system, than the western population. Also, chloroquine and hydroxychloroquine, the two most revered drugs in the context of COVID-19 treatment, have been extensively used at community level in India for the prevention and treatment of malaria.^8^ Given the immunomodulatory and antiviral properties of these drugs, their widespread usage might have contributed to defense against SARS-CoV-2.

Second, the epigenetic factors like environment and food habits might have some role in boosting immunity. Several kinds of herbs and spices like turmeric, cloves, ginger, mustard, saffron, cardamom, and garlic are essential ingredients of the Indian cuisine. These spices are rich in bioactive compounds and phytochemicals which possess medicinal properties (Sengupta et al., 2004). Turmeric (Indian saffron), a product of *Curcuma longa*, is a rhizomatous herbaceous perennial plant belonging to the ginger family that is routinely used in Indian households as a culinary spice and as a component in religious ceremonies. It has also been used as a traditional medicine over centuries. Curcumin, the bioactive compound in turmeric has been recognized for its potent antioxidant, anti-inflammatory, antimutagenic, antimicrobial, and anticancer properties. It has demonstrated antiviral activity against a wide range of viruses including Human Immunodeficiency Virus 1 and 2, Influenza Virus (H1N1, PR8, and H8N1), Herpes Simplex Virus (HSV) 1 and 2, Coxsackievirus, Hepatitis B and C viruses, Human Papillomavirus (HPV) types 16 and 18, Japanese Encephalitis virus, and Human T cell Lymphotropic Virus type 1 (Moghadamtousi et al., 2014). Similarly, ginger, another rhizomatous spice used routinely in Indian foods contains several bioactive phenolic and terpene compounds that have demonstrated antiviral activity against human respiratory syncytial virus (blocks viral attachment and internalization) (Mao et al., 2019). Garlic (*Allium sativum*), a popular condiment used in Indian foods, has been shown to possess antiviral activity against HSV-1 and -2, Parainfluenza Virus type 3, and Human Rhinovirus type 2 (Lee et al., 2016). Capsaicin, an active component of chili peppers, acts as an agonist at vanilloid receptor 1 of dendritic cells and enhances the antiviral activity of CD8 + cytotoxic T-cells by increasing MHC class I-restricted viral antigen presentation in dendritic cells (Weber et al., 1992). It has been shown to inhibit influenza A virus replication in cell cultures in a dose dependent manner (Marois et al., 2014). Even, studies from China have highlighted the possible therapeutic role of traditional Chinese medicines in the treatment of COVID-19 (Yang et al., 2020). Thus, considering the multi-pharmacological activity of the active components in Indian spices, it is possible that they might have some role in defense against SARS-CoV-2.

Third, the Indian population possesses a high genetic diversity in the immune response genes, collectively referred to as human leukocyte antigen (HLA) complex, much more extensive than the Caucasian population and this is attributed to the high microbial load to which the Indians are exposed during life (Mehra, 2010). The HLA complex serves to induce and regulate immune responses. It helps in distinguishing self antigens from non-self foreign proteins. The foreign antigens can be recognized by the T-cells only if they are presented in association with HLA proteins. More the number of HLA allotypes expressed on the cell surface, broader the range of foreign antigens they can recognize and present to the T-cells. Thus, individuals with heterozygous HLA alleles possess the ability to present a broader repertoire of antigenic peptides to the immune cells as compared to homozygotes (Mosaad, 2015). According to a study at All India Institute of Medical Sciences, New Delhi, India, several novel HLA alleles (HLA-A^∗^3303) and unique haplotypes (HLA-A2-B50-DR3) have been observed in the Indian population, which are not known to occur in other ethnic groups (Mehra, 2010). Whether such differences have any influence on the severity of SARS-CoV-2 infection need to be investigated. Recently, Nguyen et al. (2020) evaluated the role of cross-protective immunity in COVID-19 and reported that individuals with HLA-B^∗^46:01 allele are particularly vulnerable to the disease due to expression of low levels of SARS-CoV-2 binding peptides, while those with HLA-B^∗^15:03 possess an enhanced capacity to present highly conserved SARS-CoV-2 peptides that are shared among common human coronaviruses, indicating its potential to elicit cross-protective T-cell based immunity. Therefore, genetic diversity of HLA alleles can be an important factor in determining the susceptibility to SARS-CoV-2 infection.

Fourth, allelic variation in angiotensin converting enzyme 2 (ACE2) receptor can be an important factor determining the susceptibility to SARS-CoV-2 infection. Cell culture assays and molecular superimposition studies indicate that SARS-CoV-2 spike (S) protein has got high affinity for human ACE2 receptors and utilizes the same for gaining entry into the target cells. ACE2 receptors are widely distributed in body tissues, the highest expression being observed in type II alveolar epithelial cells (Hussain et al., 2020). Recent studies suggest a correlation between the level of ACE2 expression on cell membrane and viral infectivity, which in turn governs disease severity and clinical outcomes (Jia et al., 2005). Hussain et al. (2020) reported that certain allelic variants of ACE2 (S19P and E329G) demonstrates low binding affinity for the viral spike protein and may thus confer resistance to SARS-CoV-2 infection. A study by Calcagnile et al. (2020) found that two SNPs- S19P (common in Africans) and K26R (common in Europeans) can potentially affect the interaction between ACE2 and SARS-CoV-2 spike glycoprotein. While the former decreases ACE2 affinity for the spike protein and lowers the susceptibility, the latter increases the receptor affinity and predisposes to more severe disease. Hence, genetic polymorphisms of ACE2 receptors might be an important factor in determining the susceptibility to SARS-CoV-2.

Fifth, the microRNAs (miRNAs) might play a crucial role in defense against SARS-CoV-2. miRNAs are small non-coding RNAs that are thought to regulate gene expression and cell differentiation, and have the ability to inhibit viral replication in a sequence-specific manner. Considering their unique mechanism of action, miRNAs have become attractive candidates as diagnostic biomarkers and therapeutic targets (Correia et al., 2017). Several studies have highlighted the potential role of miRNAs in the regulation of antiviral defense against H1N1 influenza, HIV-1, HSV-1, HPV, enterovirus-71, hepatitis B and C, and dengue viruses (Barbu et al., 2020). A recent study by Translational Bioinformatics Group at ICGEB and Department of Biochemistry, Jamia Hamdard, New Delhi, India, revealed that nine host miRNAs can potentially target SARS-CoV-2. These are hsa-let-7a, hsa-miR101, hsa-miR125a-5p, hsa-miR126, hsa-miR222, hsa-miR23b, hsa-miR378, hsa-miR380-5 and hsa-miR98. Interestingly, a single unique miRNA, hsa-miR-27b, has been found to be specifically targeting the Indian SARS-CoV-2 genome, which has not been detected elsewhere. It has been hypothesized that hsa-miR-27b has a specific role in defense against SARS-CoV-2 in Indian population that harbors this unique sequence (Sardar et al., 2020). These findings indicate a possible link between host miRNAs and the disease severity as well as treatment outcomes.

Sixth, the Indians have a higher percentage of nature killer (NK) cells, compared to other ethnic groups (Rathore et al., 2018). It has been postulated that the development, tolerance and activation of NK cells are regulated by the killer cell immunoglobulin-like receptors (KIR) present on their surface. These receptors interact with HLA class I expressed by target cells and regulate cytolytic activity (Campbell and Purdy, 2011). While most KIRs are inhibitory and tend to suppress NK cell function, activating receptors (KIR2DS1-5, KIR3DS1) are upregulated in viral infections and host cell aberrations. All activating KIRs (except KIR2DS4) are encoded by B haplotype (Hiby et al., 2010). It has been observed that B haplotypes are more prevalent in non-Caucasian populations such as Australia Aborigines and Asian Indians than in Caucasian populations, and such populations are likely to be under high selection pressure from infectious diseases, much more than other ethnic groups. KIR genes display extraordinary level of polymorphism and diversity among different populations because of which there may be significant differences in ligand affinity or signal transduction. Population-based analysis of KIR genes reveal that KIR2DS2, an activating KIR, has high frequencies (>70%) in Australia aborigines and Indian population, but low frequencies in other ethnic groups (Middleton and Gonzelez, 2010). According to Rajalingam et al. (2008), the Indians might have acquired activating KIR genes through a process of natural selection that enabled them to survive epidemics during their pre-historic migrations from Africa. Thus, it is possible that the diversity in KIR gene complex occurred as a result of natural selection by pathogens so as to offer a survival benefit. Such diversification might be a reason for the Indian population to respond differently to SARS-CoV-2.

Seventh, live attenuated vaccines like Bacillus Calmette-Guérin (BCG) and Mumps, Measles, Rubella (MMR) commonly administered during childhood, have been found to confer non-specific protection against unrelated lethal infections by inducing “trained” non-specific innate immune cells for more efficient host response against wide range of pathogens (Fidel and Noverr, 2020). The BCG vaccine, given universally to Indian babies at birth for protection against tuberculosis, might confer some protection against SARS-CoV-2, possibly through activation of T-cell mediated immune response. BCG has no direct antiviral action, but it may act as an immunopotentiator (Tsiang et al., 1981). Studies have demonstrated the ability of BCG vaccine in reducing viraemia associated with yellow fever vaccine (Arts et al., 2018), and decreasing the severity of encephalomyocarditis virus infection in mice (Floc’h and Werner, 1976). Researchers from New York Institute of Technology correlated global COVID-19 transmission with BCG vaccine coverage data and observed that countries with a policy for universal BCG vaccination have lower number of COVID-19 cases than those without such policy, such as the United States and Italy (Miller et al., 2020). Japan, where universal BCG vaccination policy is in place since 1947, has maintained a low fatality rate, despite an early spike in COVID-19 cases. Iran, one of the countries badly hit by this pandemic, started universal BCG vaccination only in 1984, potentially leaving anyone above 36 years of age unprotected. However, according to WHO, such comparisons are prone to significant bias from many confounders, including variations in demographics and disease burden, COVID-19 testing rates and the stage of the pandemic in each country.^9^ Two randomized controlled trials, BCG-CORONA (NCT04328441) and BRACE (NCT04327206) trials are underway, evaluating the efficacy of BCG vaccine in reducing the viral load and/or sequelae associated with COVID-19. One limitation of BCG vaccination is seroconversion, which is the basis for diagnosis of TB in several countries. Therefore, MMR, another live attenuated vaccine, might be a potential option for inducing beneficial non-specific effects in human populations and thus provide protection against the catastrophic sequelae of COVID-19. A strong correlation has been observed between individuals in geographical locations who received MMR vaccine and reduced COVID-19 death rates (Gold, 2020). Interestingly, despite children being highly susceptibility to flu, very few children have been affected during the ongoing COVID-19 pandemic. Also, there has been a striking difference in the COVID-19 mortality rates between children and adults. One explanation is that children are protected owing to their recent and more frequent exposures to live attenuated vaccines (BCG, MMR, chickenpox and rotavirus) which can induce the trained myeloid-derived suppressor cells and thus limit disease severity (Fidel and Noverr, 2020). Though conclusive evidence on this ground is lacking at the moment, considering the positive correlation between the use of live attenuated vaccines and lower COVID-19 severity and death rates, hopes remain high for a possible protective action of these vaccines against SARS-CoV-2.

### Do Environmental Factors Have Any Influence on SARS-CoV-2 Transmission?

India is a vast country with varied weather conditions. The western part of the country is known for hot dry summers with temperatures exceeding 50°C, while the Himalayan belt in the northern part records temperatures as low as −30°C. Previous studies have shown the impact of weather variables on the transmission dynamics of diseases like influenza and SARS. Transmission of influenza is facilitated in the presence of cold and/or dry air. A sharp rise in influenza virus activity in northern Europe during 2010–2018 was correlated with low temperature and low ultraviolet (UV) radiation indices. Based on these facts, it was assumed that COVID-19 transmission might decrease or even disappear with the rise in ambient temperature and UV radiation during the summer months. However, a study by Yao et al. (2020) incorporating meteorological data and basic reproduction number (R_0_) of the virus from 224 Chinese cities revealed that high temperature, humidity and UV radiation has no significant impact on cumulative incidence rate and transmission of SARS-CoV-2. Infact, countries like Iran and Australia continued to have rapid disease transmission and exponential rise in the number of cases despite high temperature and humidity, indicating that the inverse relationship between ambient temperature and COVID-19 transmission lacks rationality. Studies have shown that an interplay between macrodomain (MAC) and papain-like protease 2 (PLP2) domain of coronavirus NSP3 impacts viral replication, host innate immune antagonism and virulence. Strains harboring mutations in these two domains are more rapidly degraded at non-permissive temperatures (37°C and 40°C) than the wild-type strains (Deng et al., 2019), indicating that higher temperatures may lower the virulence and pathogenicity of temperature-sensitive SARS-CoV-2 mutants.

## Discussion

The COVID-19 fatality rate in India is among the lowest in the world (2.8% against a global average of 4.7%). Also, the disease has been observed to be less severe among Indians with a significantly higher recovery rate (60.9% versus a global average of 56.6%) and doubling time (20.3 days) as compared to the populations elsewhere.^3,4, 10^ Strict lockdown measures implemented nationwide for more than two months might have kept the transmission and deaths in check. This must have contributed significantly to the much desired flattening of the epidemic curve. There are speculations that India’s predominantly young population as compared to the western world (with a higher proportion of elderly people), and the presence of a less virulent strain of the virus in circulation are helping keep fatalities low in India. However, there are no evidences in support of these claims. In fact, phylogenetic analysis has revealed that four clades of the virus: O (ancestral clade from China), B (from China), A3 (from Iran) and A2a (from Iran, Europe, and other countries) are in circulation in India with A2a being the dominant type (Biswas and Majumder, 2020). There are reports that the official death counts are much lower as compared to the actual death toll. On April 18, 2020, China revised the death toll to 4632 with a 50% spike in fatality figures.^11^ Such discrepancies may arise because the official count considers only the hospital deaths, while majority of the deaths still happen at home. Also, death due to COVID-19 may not be reported in presence of other comorbidities. Tracking deaths is far more reliable than cases, which are significantly affected by testing bias. Roughly, 86% of the COVID-19 patients are asymptomatic who hardly get tested and serve as potential source for transmission of infection. In a densely populated country like India, mass screening of population for COVID-19 is not feasible. So there are chances that India might be missing some deaths and not diagnosing every case correctly. However, considering the magnitude of the pandemic, India has ramped-up the testing capacity with nearly 250,000 samples being screened and over 20,000 active cases being detected daily.^6^ Still, the fatality rate in Indian cases is low and none has got a definite answer for that. While this apparent protection among Indians is largely attributed to non-heritable influences as discussed earlier, a safe and effective vaccine against SARS-CoV-2 can reduce disease severity, control transmission, and prevent future infections across all populations. Vaccines function by inducing both arms of the adaptive immune response: humoral immunity (producing neutralizing antibodies that prevent virus attachment) and cell-mediated immunity (activating antigen presenting cells, T-cells, and NK cells that recognize and kill virus-infected cells) (Mukherjee, 2020). There are currently ten vaccines undergoing clinical trials against COVID-19 based on several technologies, such as, live attenuated, inactivated, mRNA, DNA, viral vector, and peptide subunit based platforms (Mullard, 2020). A strong global vaccination program with equitable distribution of promising vaccine candidates to all affected regions is needed to tackle the pandemic. This perspective puts forward several hypotheses which may explain individual and population susceptibility to COVID-19 which in turn, may help prioritize vaccination in high risk individuals and groups, and thus reduce morbidity and mortality across populations. Whether the above factors generate sufficient evidence to provide satisfactory explanation for their apparent protective effects on Indian population during COVID-19 pandemic, will be clear as the situation unfolds further.

## Data Availability Statement

The original contributions presented in the study are included in the article/supplementary material, further inquiries can be directed to the corresponding author.

## Author Contributions

AS and RG: conceptualization, data curation, and methodology. AS, RG, and VN: supervision. AS, RG, VN, and SM: validation, and visualization. AS and RG: writing – original draft. AS, RG, VN, and SM: writing – review and editing. All authors contributed to the article and approved the submitted version.

## Conflict of Interest

The authors declare that the research was conducted in the absence of any commercial or financial relationships that could be construed as a potential conflict of interest.

## Footnotes

1 World Health Organization (WHO) Emergency Committee. Statement on the second meeting of the International Health Regulations (2005) Emergency Committee regarding the outbreak of novel coronavirus (2019-nCoV). Geneva: WHO; 30 January 2020. Available from: https://www.who.int/news-room/detail/30-01-2020-statement-on-the-second-meeting-of-the-international-health-regulations-(2005)-emergency-committee-regarding-the-outbreak-of-novel-coronavirus-(2019-ncov) (Accessed May 25, 2020).

2 World Health Organization (WHO) Emergency Committee. WHO Director-General’s opening remarks at the media briefing on COVID-19 – 11 March 2020. Geneva: WHO; 11 March 2020. Available from: https://www.who.int/dg/speeches/detail/who-director-general-s-opening-remarks-at-the-media-briefing-on-covid-19—11-march-2020 (Accessed May 25, 2020).

3 World Health Organization (WHO). Coronavirus disease (COVID-19): situation report, 165. Geneva: WHO; 03 July 2020. Available from: https://www.who.int/emergencies/diseases/novel-coronavirus-2019/situation-reports/ (Accessed July 3, 2020).

4 Available from: https://www.hindustantimes.com/india-news/only-4-16-of-covid-19-patients-in-india-require-ventilator-support-official/story-isRJJrcO3mGdK8iHtd4w3H.html (Accessed July 3, 2020).

5 Available from: https://www.indiatoday.in/india/story/wuhan-s-l-strain-may-be-behind-gujarat-s-high-death-rate-experts-1671346-2020-04-26 (Accessed May 25, 2020).

6 Available from: https://www.mohfw.gov.in/ (Accessed July 03, 2020).

7 Available from: https://ourworldindata.org/grapher/total-covid-deaths-per-million (Accessed May 25, 2020).

8 Available from: https://theprint.in/india/indians-seem-to-have-genetic-and-regional-advantages-in-fight-against-coronavirus/393939/ (Accessed May 25, 2020).

9 World Health Organization (WHO). Scientific brief: Bacille Calmette-Guérin (BCG) vaccination and COVID-19. Geneva: WHO; 12 April 2020. Available from: https://www.who.int/news-room/commentaries/detail/bacille-calmette-gu%C3%A9rin-(bcg)-vaccination-and-covid-19 (accessed May 25, 2020).

10 Available from: https://www.hindustantimes.com/india-news/another-grim-milestone-india-no-3-in-total-cases/story-lJPkEouOjojcdp6HrxizVI.html (Accessed July 5, 2020).

11 Available from: https://economictimes.indiatimes.com/news/international/world-news/covid-19-chinas-imported-cases-rise-to-1566-revised-death-toll-4632/articleshow/75216036.cms (Accessed May 25, 2020).
